# Adjunctive accelerated repetitive transcranial magnetic stimulation for older patients with depression: A systematic review

**DOI:** 10.3389/fnagi.2022.1036676

**Published:** 2022-10-24

**Authors:** Wei Zheng, Xin-Yang Zhang, Rui Xu, Xiong Huang, Ying-Jun Zheng, Xing-Bing Huang, Ze-Zhi Li, Huo-Di Chen

**Affiliations:** ^1^The Affiliated Brain Hospital of Guangzhou Medical University, Guangzhou, China; ^2^Laboratory of Laser Sports Medicine, School of Sports Science, South China Normal University, Guangzhou, China

**Keywords:** accelerated TMS, depression, systematic review, older patients, response

## Abstract

**Objective:**

We performed this systemic review to investigate the therapeutic potential and safety of adjunctive accelerated repetitive transcranial magnetic stimulation (aTMS) for older patients with depression.

**Methods:**

We included published randomized clinical trials (RCTs) and observational studies targeting adjunctive aTMS for older patients with depression.

**Results:**

Two open-label self-controlled studies (*n* = 29) fulfilled the criteria for inclusion. The included studies reported significant improvements in depressive symptoms from baseline to post-aTMS (all *Ps* < 0.05). One study reported a dropout rate of 10.5% (2/19). Mild headache was the most common adverse reaction.

**Conclusion:**

The currently available evidence from two open-label self-controlled studies indicates that adjunctive aTMS is a safe and effective therapy for older patients with depression.

## Introduction

Depression is a leading cause of disability (World Health Organization, 2017), and occurs in 7% of the elderly population worldwide (World Health Organization, 2016). A diagnosis of depression in old age is often associated with poorer long-term prognoses, higher recurrence rates, lower quality of life, and a greater likelihood of morbidity and early mortality (Mitchell and Subramaniam, 2005; Aziz and Steffens, 2013). Up to 1/3 of individuals experiencing major depressive disorder (MDD), particularly in the elderly population, fail to achieve clinical remission after acute pharmacological treatment (Rush et al., 2006). Because comorbid physical diseases are common, elderly patients with depression are highly likely to experience side effects of medication (Kok and Reynolds, 2017). Thus, non-pharmacological treatments, such as electroconvulsive therapy (ECT) (Dong et al., 2018; Jiang et al., 2020), transcranial magnetic stimulation (TMS) (Blumberger et al., 2015; Conelea et al., 2017), transcranial direct current stimulation (tDCS) (Kumar et al., 2020; Brooks et al., 2021), vagus nerve stimulation (VNS) (van Rooij et al., 2020), deep brain stimulation (DBS) (McDonald, 2016) and theta-burst stimulation (TBS) (Cristancho et al., 2020), may be reasonable alternatives for older patients with depression.

A type of non-invasive brain stimulation, repetitive transcranial magnetic stimulation (rTMS), was approved by the FDA as a treatment for MDD in 2008 (Holtzheimer et al., 2010). A network meta-analysis of 81 randomized clinical trials (RCTs) found that active rTMS showed a significantly higher clinical response and remission rates than non-active rTMS (Brunoni et al., 2017). A typical course for rTMS involves five days of treatment/week over a period of 3–6 weeks (Holtzheimer et al., 2010). However, this schedule may be inconvenient for patients and can hinder compliance (Frey et al., 2020). Thus, consolidating the treatment (e.g., over 2–3 days) may make it more accessible and could potentially increase compliance.

Accelerated rTMS (aTMS) protocols have been studied as a potential solution for this problem (Sonmez et al., 2019). Recent meta-analyses have found that aTMS protocols may be effective for individuals suffering from depression (Sonmez et al., 2019) and post-stroke depression (PSD) (Frey et al., 2020). A randomized controlled study (RCT) of twice-daily rTMS for the treatment of MDD found that rTMS given twice daily was effective and safe (Loo et al., 2007). Two open-label studies have also reported positive findings for adjunctive aTMS as a therapy in addition to antidepressants for older patients with depression (Dardenne et al., 2018; Desbeaumes Jodoin et al., 2019). For example, Dardenne et al. reported that aTMS was safe and well-tolerated in older patients with MDD (≥65 years old) (Dardenne et al., 2018). Similarly, a recent study reported that aTMS protocol (two sessions per day) is a safe and effective treatment for older patients (≥60 years old) suffering from treatment-resistant depression (TRD) (Desbeaumes Jodoin et al., 2019).

To date, no systematic review examining the therapeutic role and safety of adjunctive aTMS for older patients with depression has been published. In view of this important gap, we conducted this review to systematically investigate the efficacy and safety of adjunctive aTMS for older patients with depression.

## Methods

### Search strategy and selection criteria

Two investigators (X-YZ and RX) independently searched electronic databases (including PsycINFO, Cochrane Library, PubMed, EMBASE, Chinese Journal Net, and WanFang) and manually checked reference lists of the included studies (Dardenne et al., 2018; Desbeaumes Jodoin et al., 2019) and relevant reviews (Mutz et al., 2019; Sonmez et al., 2019) for eligible studies on adjunctive aTMS for older patients with depression. The initial search was completed by two investigators (XYZ and RX) on December 16, 2021, using the following search terms: (accelerated TMS OR accelerated rTMS OR aTMS OR accelerated transcranial magnetic stimulation OR accelerated repetitive transcranial magnetic stimulation) AND (depression OR depressed OR depressive) AND (aged OR elderly OR older adult OR aging).

In line with PRISMA guidelines (Moher et al., 2009), we included studies that fulfilled the following ***PICOS*** criteria. ***P***articipants: older patients (≥60 years old) suffering from uni- or bi-polar depression, as defined by the respective studies. ***I***ntervention *vs*. ***C***omparison: real aTMS with antidepressants *vs*. antidepressant monotherapy or sham aTMS wiht antidepressants; aTMS added to antidepressants (observational studies). ***O***utcomes: the primary outcome was changed in depressive symptoms as measured by depression scales [i.e., the Montgomery-Asberg Depression Rating Scale (MADRS) (Montgomery and Asberg, 1979; Zhong et al., 2011)]. Key secondary outcomes reported in this systematic review were study-defined response and remission, dropout rate, and adverse events. ***S***tudy: only published RCTs or observational studies (single-group, before-after design) investigating the efficacy and safety of aTMS in combination with antidepressants for older patients with uni- and bi-polar depression were eligible for inclusion. As reported previously (Mutz et al., 2019), TBS included the following three different treatment strategies: intermittent TBS, continuous TBS, or bilateral TBS. Thus, studies with at least two rTMS sessions rather than one TBS session per day were included. Review articles, retrospective studies, and case reports/series were excluded.

### Data extraction

Two independent investigators (X-YZ and RX) extracted relevant data from each included study. Any disagreements were resolved through consensus or, if needed, through discussion with the senior author (WZ). Missing data were requested by contacting first and/or corresponding authors and/or searching for the data from other reviews (Sonmez et al., 2019).

### Quality assessment

The quality of RCT were independently evaluated by two investigators (X-YZ and RX) using the Cochrane risk of bias (Higgins et al., 2011).

## Results

### Study selection

**Figure 1:**
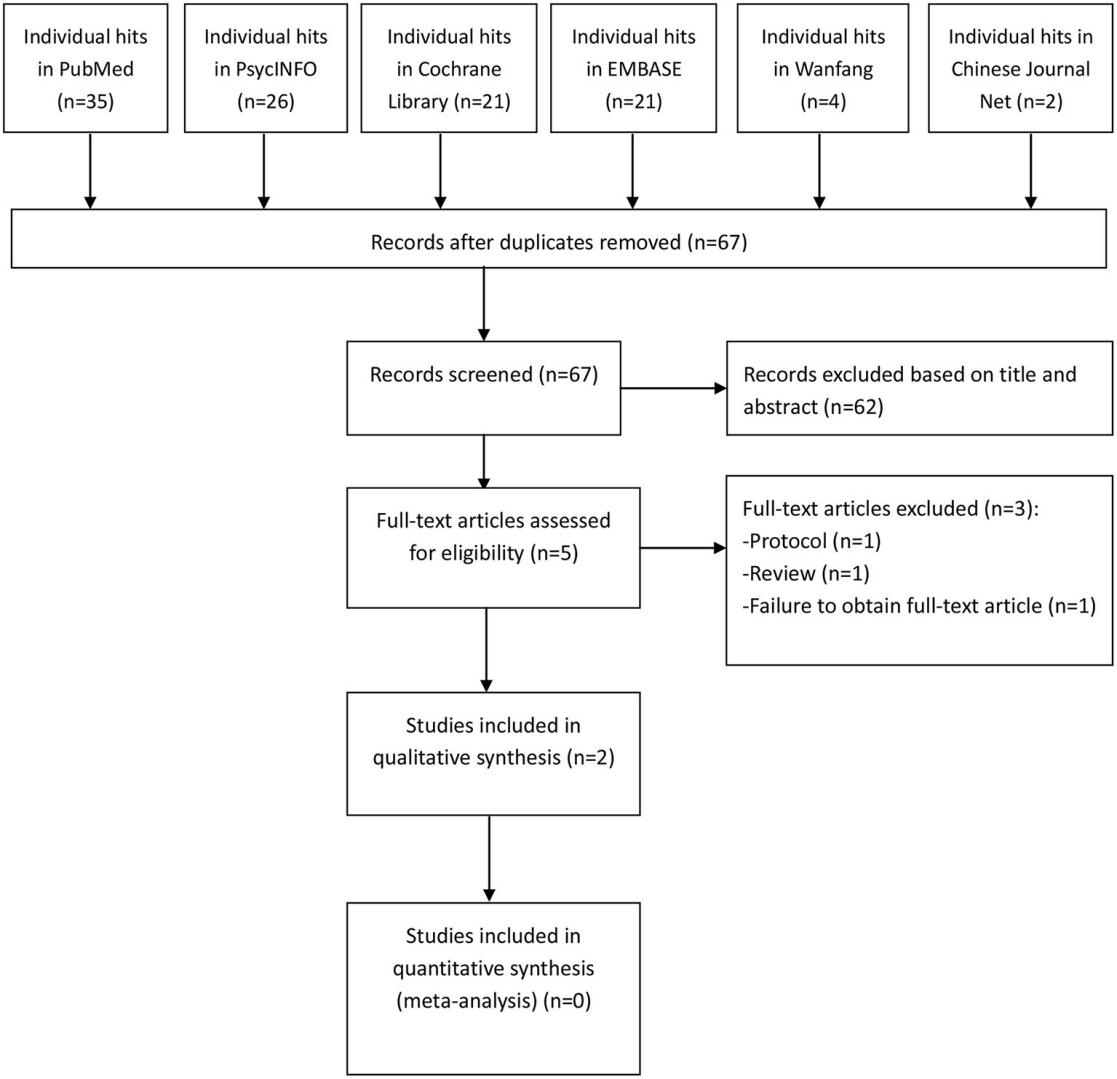
PRISMA flow diagram.

As shown in Figure 1, we identified a total of 109 hits in this systematic review. In the end, two open-label self-controlled studies met the inclusion criteria and were included in our qualitative analysis (Dardenne et al., 2018; Desbeaumes Jodoin et al., 2019).

### Study characteristics

The characteristics of the two observational studies (Dardenne et al., 2018; Desbeaumes Jodoin et al., 2019), covering 29 older patients with MDD (*n* = 27) or bipolar depression (*n* = 2), are summarized in Table 1. One of the studies (*n* = 10) (Dardenne et al., 2018) was conducted in Belgium; the other (*n* = 19) (Desbeaumes Jodoin et al., 2019) was conducted in Canada.

**Table 1:** Summary of characteristics of included studies.

**References (country)**	***N*** **(♂/♀)**	**Study design**	**- Diagnosis - Diagnostic criteria**	**Age: yrs (range)**	**Medication status**	**Type site; Frequency (intensity)**	**Total stimuli (stimuli/session); - Total days (sessions/day, Total sessions)**	**- Trains/ session - Train length- Intertrain**
Dardenne et al. (2018) (Belgium)	10 (0/10)	Open-label	- MDD - DSM-IV	73.9 (65–82)	Psychotro pic -allowed	HF-rTMS L-DLPFC; 20 Hz (110%)	31200 (1560 pluses); 4 (5, 20)	−39 s - 2 s - 12 s
Desbeaumes Jodoin et al. (2019) (Canada)	19 (10/9)^a^	Open-label	-TRD (17 unipolar, 2 bipolar) - DSM-5	71.0 (60–89)	Psychotro pic -allowed	HF-rTMS L-DLPFC; 20 Hz (110%)	60000–90000 (3000 pluses); 10–15 (2, 20–30)	-150 s -5 s -25 s

a Data were extracted only focusing on older patients (≥60 years old) with depression.

DSM, Diagnostic and Statistical Manual of Mental Disorders; HF, high frequency; L-DLPFC, left dorsolateral prefrontal cortex; MDD, major depressive disorder; N, number of patients; NR, not reported; NOS, newcastle-ottawa scale; rTMS, repetitive transcranial magnetic stimulation; TRD, treatment-resistant depression.

♂ = Male; ♀ = Female.

### Assessment of study quality

We did not use the Cochrane risk of bias assessment because no RCTs fulfilled the inclusion criteria.

### Depressive symptoms

As shown in Table 2, the two included studies consistently reported significant improvements in depressive symptoms from baseline to post-aTMS (all *Ps* < 0.05). In Dardenne et al.'s (2018) study, 40% (4/10) of older patients with MDD showed responses, and 20% (2/10) met the remission criteria. In Desbeaumes Jodoin, Miron and Lespérance (2019) study, 14 out of 19 older patients (73.7%) responded to aTMS, and 63.2% (12/19) met remission criteria.

**Table 2:** The improvement of depressive symptoms after aTMS.

**References**	**Assessment tools**	**At baseline (mean ±SD, *n*)**	**At end of study (mean±SD, n)**	***P-value***
Dardenne et al. (2018)	HDRS scores	22.6 ± 4.1 (*n* = 10)	10.6 ± 7.9 (*n* = 10)^a^	**0.004**
	BDI scores	25.9 ± 7.0 (*n* = 10)	10.8 ± 7.1 (*n* = 10)^a^	**0.004**
Desbeaumes Jodoin et al. (2019)	MADRS scores	21.7 ± 9.3 (*n* = 19)	9.4 ± 7.6 (*n* = 19)^b^	**<0.001**

a Patients were assessed at post-aTMS.

b Patients were assessed at seven days after the last aTMS session.

Bolded values are *P* < 0.05.

aTMS, accelerated transcranial magnetic stimulation; BDI, Beck Depression Inventory; HDRS, Hamilton Depression Rating Scale; MADRS, Montgomery-Asberg Depression Rating Scale; n, number of patients.

### Dropout rate and adverse events

Dropout rate and adverse events are summarized in Table 3. The dropout rate was 10.5% (2/19) in Desbeaumes Jodoin, Miron and Lespérance (2019) study and 0% (0/10) in Dardenne et al.'s (2018) study. Mild headaches were the most common adverse events, accounting for 40% of side effects (Dardenne et al., 2018).

**Table 3:** Dropout rate and adverse events.

**References**	**Sample size**	**Dropout rate**	**Adverse events**	
		**Total (%)**	**Events**	**Total (%)**
**Observational studies (*****n*** **= 29)**				
Dardenne et al. (2018)	10	0 (0)	Local discomfort	1 (10)
			Mild headache	4 (40)
Desbeaumes Jodoin et al. (2019)	19	2 (10.5)	Headache	3 (15.8)
			Local sensitivity	3 (15.8)
			Fatigue	1 (5.3)

## Discussion

This article is the first systematic review to examine the potential therapeutic role and safety of adjunctive aTMS for older patients (≥60 years old) suffering from depression. Only two observational studies (Dardenne et al., 2018; Desbeaumes Jodoin et al., 2019) involving 29 older patients with depression were included in this systematic review. The two studies (Dardenne et al., 2018; Desbeaumes Jodoin et al., 2019) were published within the last three years, indicating that this is a novel and clinically important topic. This systematic review provides preliminary support for the utility of aTMS for reducing depressive symptoms in older patients with depression. Furthermore, adjunctive rTMS was safe and well-tolerated in elderly depressed patients. However, aTMS may have resulted in higher discomfort rates than standard daily rTMS (Fitzgerald et al., 2018).

According to this systematic review, adjunctive aTMS appears to be effective in treating older patients suffering from depression, although the long-term efficacy was not reported. The rationale for an accelerated approach comes from the idea that repeated application of stimulation within short time intervals could be associated with greater antidepressant effects (Sonmez et al., 2019). A recent review reported that high-frequency (HF) rTMS delivered over the left dorsolateral prefrontal cortex (DLPFC) could reduce suicidal behavior in individuals with the treatment-resistant depression (Godi et al., 2021).

The response rates of HF rTMS tended to range from 20 to 30% (O'Reardon et al., 2007; Avery et al., 2008; George et al., 2010), which was far lower than the response rate to aTMS (73.7%) (Desbeaumes Jodoin et al., 2019). However, a recent RCT, involving 115 outpatients with MDD who randomly received either aTMS or standard daily rTMS, found that aTMS and rTMS had comparable efficacy for treating depression (Fitzgerald et al., 2018). Although this systematic review found that aTMS may be an effective therapy in elderly patients with depression, a variety of parameters have been applied to the two included studies (Dardenne et al., 2018; Desbeaumes Jodoin et al., 2019). For example, the total stimuli of aTMS ranged between 31,200 and 90,000, and the optimal parameters for aTMS remain unclear.

The following limitations must be considered. First, only two open-label self-controlled studies (single-group, before-after design) examining the efficacy and safety of adjunctive aTMS for older patients with depression were included (Dardenne et al., 2018; Desbeaumes Jodoin et al., 2019). Second, the relatively small sample sizes in both studies potentially reduced their power and increased the possibility of type II error. Third, this systematic review on adjunctive aTMS for older patients with depression has not been registered. Fourth, given that the heterogeneity between the studies, a quantitative analysis could not be performed in this study. Finally, some important outcome measures, such as cognitive functioning, were not reported in the included studies.

## Conclusions

The current evidence from open-label self-controlled studies, while limited, indicates that adjunctive aTMS is a safe and effective therapy for older patients with depression. Further RCTs with rigorous methodology need to be performed in order to confirm and extend these findings.

## Data availability statement

The original contributions presented in the study are included in the article/supplementary material, further inquiries can be directed to the corresponding authors.

## Author contributions

X-YZ and RX selected studies and extracted the data. WZ reviewed all the data, helped mediate disagreements, and wrote the first draft. All authors contributed to the interpretation of data and approved the final manuscript.

## Funding

This study was funded by the the Science and Technology Planning Project of Liwan District of Guangzhou (202004034), National Natural Science Foundation of China (82101609), Scientific Research Project of Guangzhou Bureau of Education (202032762), Science and Technology Program Project of Guangzhou (202102020658), Guangzhou Health Science and Technology Project (20211A011045), Guangzhou science and Technology Project of traditional Chinese Medicine and integrated traditional Chinese and Western medicine (20212A011018), China International Medical Exchange Foundation (Z-2018-35-2002), Guangzhou Clinical Characteristic Technology Project (2019TS67), science and Technology Program Project of Guangzhou (202102020658), and Guangdong Hospital Association (2019ZD06). The funders had no role in study design, data collection and analysis, decision to publish, or preparation of the manuscript.

## Conflict of interest

The authors declare that the research was conducted in the absence of any commercial or financial relationships that could be construed as a potential conflict of interest.

## Publisher's note

All claims expressed in this article are solely those of the authors and do not necessarily represent those of their affiliated organizations, or those of the publisher, the editors and the reviewers. Any product that may be evaluated in this article, or claim that may be made by its manufacturer, is not guaranteed or endorsed by the publisher.
